# Decreased Expression and Uncoupling of Endothelial Nitric Oxide Synthase in the Cerebral Cortex of Rats with Thioacetamide-Induced Acute Liver Failure

**DOI:** 10.3390/ijms22136662

**Published:** 2021-06-22

**Authors:** Krzysztof Milewski, Anna Maria Czarnecka, Jan Albrecht, Magdalena Zielińska

**Affiliations:** Department of Neurotoxicology, Mossakowski Medical Research Institute, Polish Academy of Sciences, 5 Pawińskiego Str, 02-106 Warsaw, Poland; kmilewski@imdik.pan.pl (K.M.); aczarnecka@imdik.pan.pl (A.M.C.); jalbrecht@imdik.pan.pl (J.A.)

**Keywords:** acute liver failure, nitric oxide synthase uncoupling, cerebral blood flow, tetrahydrobiopterin

## Abstract

Acute liver failure (ALF) is associated with deregulated nitric oxide (NO) signaling in the brain, which is one of the key molecular abnormalities leading to the neuropsychiatric disorder called hepatic encephalopathy (HE). This study focuses on the effect of ALF on the relatively unexplored endothelial NOS isoform (eNOS). The cerebral prefrontal cortices of rats with thioacetamide (TAA)-induced ALF showed decreased eNOS expression, which resulted in an overall reduction of NOS activity. ALF also decreased the content of the NOS cofactor, tetrahydro-L-biopterin (BH4), and evoked eNOS uncoupling (reduction of the eNOS dimer/monomer ratio). The addition of the NO precursor L-arginine in the absence of BH4 potentiated ROS accumulation, whereas nonspecific NOS inhibitor L-NAME or EDTA attenuated ROS increase. The ALF-induced decrease of eNOS content and its uncoupling concurred with, and was likely causally related to, both increased brain content of reactive oxidative species (ROS) and decreased cerebral cortical blood flow (CBF) in the same model.

## 1. Introduction

Hepatic encephalopathy (HE) is a neuropsychiatric disorder associated with acute (ALF) or chronic liver failure. Brain edema and impaired cerebral blood flow (CBF) are the two major manifestations of HE [1,2,3]. These two interrelated impairments are associated with the induction of oxidative and nitrosative stress (ONS) triggered by different stimuli. The excessive formation of reactive oxygen species (ROS) in HE-affected brain has a variety of consequences for the genome and proteome, as documented by in vitro and in vivo studies [4,5]. While N-methyl-D-aspartate (NMDA) receptor-mediated accumulation of intracellular calcium and neuroinflammation clearly appear to induce ONS in HE [5,6,7], ROS generation is also associated with the excessive production of superoxide anion (O_2_^−^), resulting from the p47phox subunit of the NADPH oxidase enzyme, as shown in cultured rat astrocytes and cortical mouse brain slices [8]. Beside the activation of oxidizing enzymes that directly produce oxygen radicals, oxidative stress also leads to the generation of nitric oxide (NO) by nitric oxide synthases (NOSs) [9]. However, being the source of reactive nitrogen species (RNS), NOS isozymes, if uncoupled, generate O_2_^−^, and therefore, will support direct oxidation [10]. The contribution of NOS isoforms to ALF-induced alterations in brain metabolism is still not entirely clear. Present knowledge of the responses of the endothelial isoform of NOS, eNOS, which is a key player in the regulation of CBF [11], lags behind that of the other isoforms. NO production altered by eNOS is expected to modulate the magnitude of ONS in the whole brain and, specifically, brain vasculature homeostasis. In theory, alterations in eNOS activity in ALF-affected brain may be linked to either reduced eNOS protein content and/or to the “uncoupling” of the enzyme by adverse regulation of well-defined “redox switches” in the eNOS enzyme or up-/down-stream signaling molecules [12]. Previously, eNOS uncoupling and subsequent oxidative stress were recorded in a cirrhotic liver, and were associated with a decreased level of the eNOS cofactor, tetrahydro-L-biopterin (BH4) [13]. Additionally, BH4 supplementation improved the endothelial dysfunction of cirrhotic liver [14,15]. It therefore appeared to be worthwhile to analyze the eNOS status in ALF-affected brain. Additional questions addressed in this study are whether and to what degree ALF-induced changes affect NO production and CBF in the whole brain.

## 2. Results

### 2.1. Rat Model of Acute Liver Failure 

**Figure 1:**
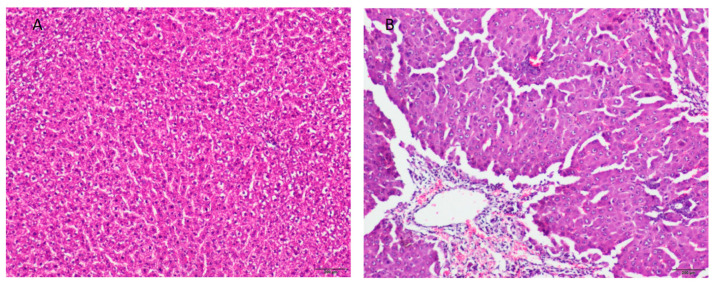
TAA-induced changes in hepatic histopathology. Micrographs of hematoxylin-eosin stained liver sections from control (**A**) and TAA-treated (**B**) rats. The histology studies of the control liver showed the normal histological appearance of liver tissue (**A**), whereas the TAA-treated liver section revealed widespread intracellular vacuolization, centrilobular bridging hepatocellular necrosis, and infiltration of inflammatory cells (**B**). Scale bars = 200 μm. Plasma level of ammonia (NH_3_) and activities of control and TAA-treated rats.

TAA-induced ALF is a commonly used animal model of HE [16,17,18,19]. Twenty-four hours after the third injection of TAA (96th h of the experiment), the rats showed visible symptoms of ALF with HE: reduced motor function with stupor or precoma state, and decreased food intake, resulting in 15–20% body weight loss in comparison to control animals (Table 1). The mortality rate was ~10%, which, in the spectrum of different ALF rat models, is considered to be moderate [20,21]. Blood plasma biochemical tests revealed a significant increase of ammonia concentration and elevated levels of liver enzymes in TAA-treated rats (Table 1). Hematoxylin and eosin staining revealed decomposition of hepatic parenchyma in TAA-treated rats, with corresponding inflammatory infiltration and massive necrosis of hepatocytes (Figure 1).

### 2.2. The Expression of NOS Isoforms in the Liver and Prefrontal Cortex

**Figure 2:**
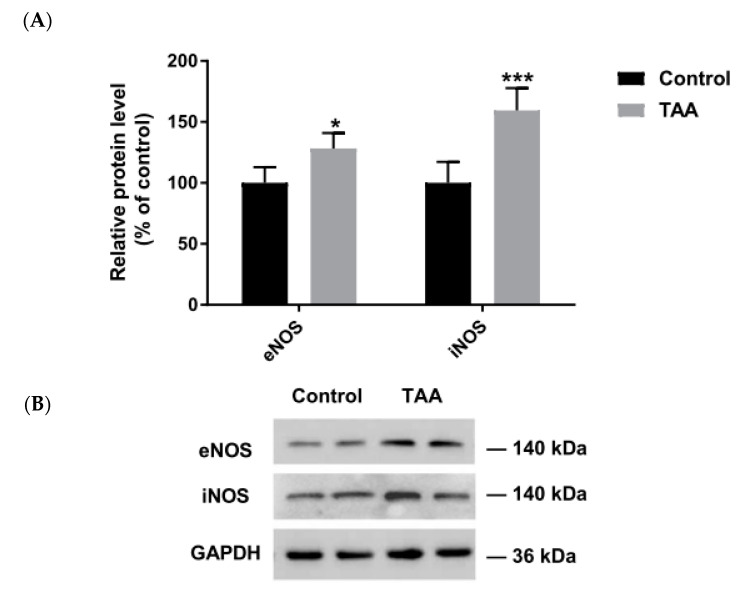
TAA treatment induces eNOS and iNOS protein level in liver. The relative protein level of control and TAA rats (**A**). Results are mean ± SD (n = 5). Significant difference vs. control * *p* < 0.05; *** *p* < 0.001. Immunostaining of eNOS and iNOS in the liver (**B**).

**Figure 3:**
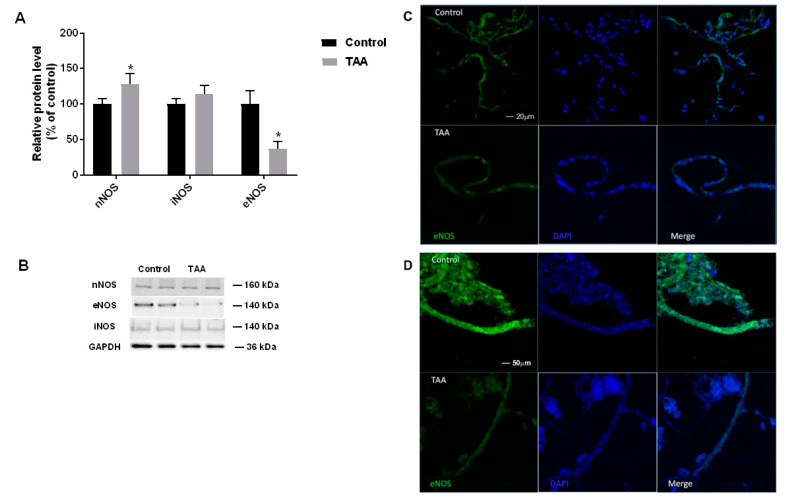
TAA induces eNOS depletion in rat brain cortex. Left panel (**A**,**B**) Western blot analysis of the relative protein level of nNOS, iNOS and eNOS in prefrontal cortex of control and TAA rats. Results are mean ± SD (n = 5) Significant difference vs. control * *p* < 0.05. Right panel (**C**,**D**) confocal microscopy images of isolated rat brain capillaries (**C**) and microvessels (**D**) of control and TAA rats. Immunofluorescence staining for eNOS. Nuclei were counterstained with DAPI (blue).

Increased expression of eNOS and iNOS was detected in the liver of TAA-treated rats (Figure 2); this result for eNOS was in line with an earlier observation made under an identical TAA treatment protocol [22]. By contrast, in the prefrontal cortex of TAA rats, a selective ~60% decrease of eNOS protein was observed, whereas nNOS and iNOS protein levels remained unchanged (Figure 3).

Strong immunofluorescence staining of eNOS was observed in control brain capillaries and microvessels (Figure 3), whereas lower immunostaining intensity was present in the vasculature isolated from TAA rat brain, where the enzyme was heterogeneously localized (Figure 3).

### 2.3. NOx Measurement and Nitration of Plasma and Prefrontal Cortex Proteins in TAA Rats

**Figure 4:**
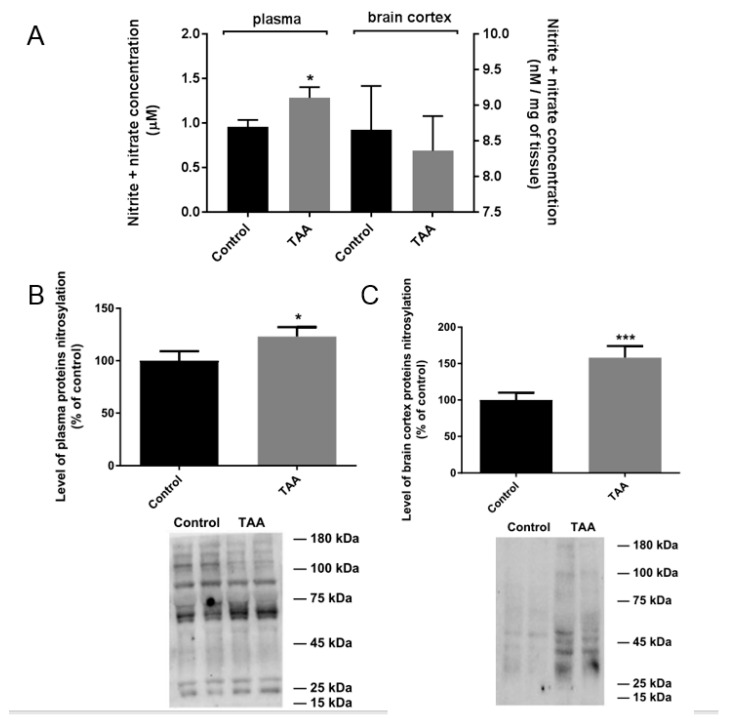
NOx (nitrite + nitrate) concentration in plasma and brain prefrontal cortex of control and TAA rats (**A**). Results are mean ± SD (n = 5) Significant difference vs. control * *p* < 0.05. Western blot analysis of protein nitration in rat blood plasma (**B**) and prefrontal cortex (**C**) of control and TAA rats. Results are mean ± SD (n = 5). Significant difference vs. control * *p* < 0.05; *** *p* < 0.001.

The NOx concentration was increased in plasma but not in the prefrontal cortex of TAA rats (Figure 4). In line with reports of increased NOS activity as a hallmark of acute liver damage [23], NOx level accompanies increased nitration of blood plasma proteins in TAA rats (Figure 4). This correlation is a well-established consequence of ONS induction and elevated NOS activity in organs of experimental animals and humans affected by different degenerative and/or inflammatory diseases [24,25,26], including human HE [27]. Accordingly, TAA treatment increased the total brain prefrontal cortical level of nitrated proteins (Figure 4).

### 2.4. eNOS Dimer/Monomer Ratio Status and BH4 Level in the Brain Prefrontal Cortex

**Figure 5:**
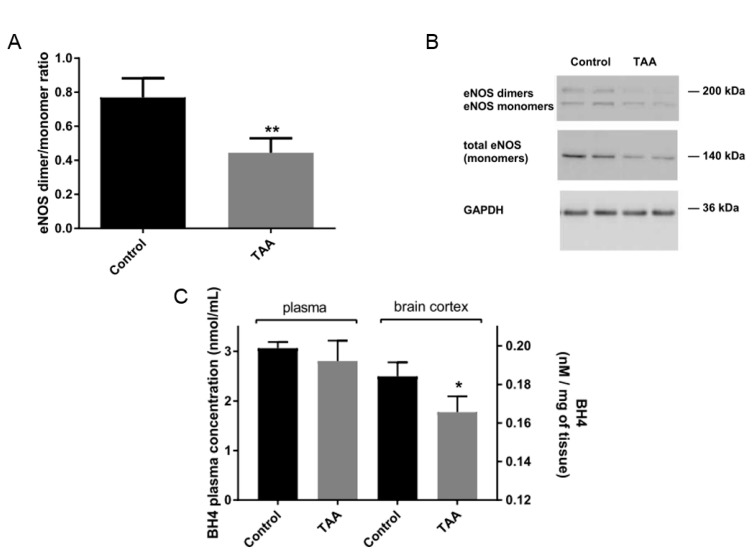
TAA impairments brain eNOS coupling. Western blot analysis of eNOS dimer/monomer ratio in rat prefrontal cortex of control and TAA rats (**A**). Results are mean ± SD (n = 5). Significant difference vs. control ** *p* < 0.01. Immunostaining of eNOS dimmers and monomers (**B**). Measurement of BH4 concentration in rat blood plasma and prefrontal cortex of control and TAA rats (**C**). Results are mean ± SD (n = 5) Significant difference vs. control * *p* < 0.05.

Both the total level of eNOS dimers and the eNOS dimer/monomer ratio were lowered in the prefrontal cortex of TAA-treated animals, suggesting that eNOS uncoupling, in addition to decreased eNOS protein, is another eNOS-related brain abnormality in ALF (Figure 5).

In TAA-treated rats, the level of NOS cofactor BH4 was not significantly affected in blood plasma, but was decreased by ~60% in the prefrontal cortex (Figure 5).

### 2.5. The Relative Contribution of NOS Isoforms to ROS Production in the Prefrontal Cortex

**Figure 6:**
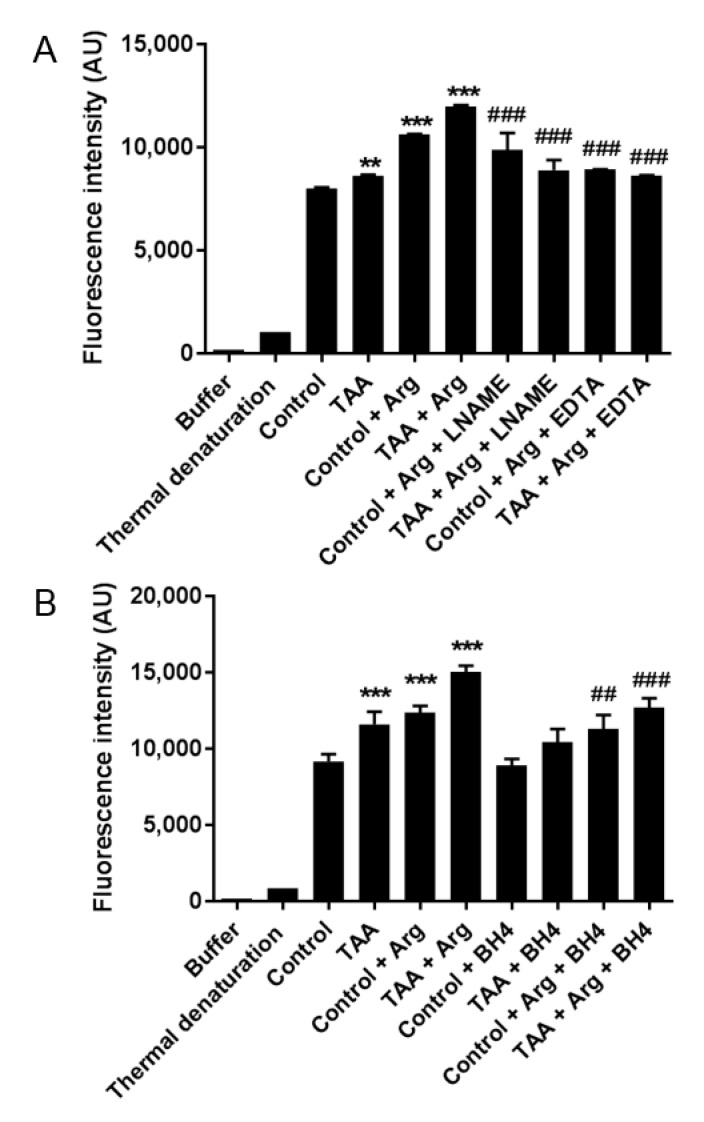
Uncoupled eNOS participates in TAA dependent enhancement of ROS production in the brain. Measurement of total ROS production in TAA rat brain cortex homogenate: effect of L-NAME and EDTA (**A**); effect of BH4 (**B**). Results are mean ± SD (n = 5). Significant difference vs. control ** *p* < 0.01; *** *p* < 0.001; ^##^ *p* < 0.01; ### *p* < 0.001 vs. corresponding (+Arg) group.

The total ROS production was elevated by ~20% in the prefrontal cortex of TAA rats (Figure 6). The addition of NOS substrate L-arginine significantly enhanced ROS production in both control and TAA rat brain homogenates, whereas nonspecific NOS inhibitor L-NAME or EDTA abolished ROS increase, underscoring the NOS-dependent nature of ROS generation. The addition of BH4, which is the main cofactor contributing to NOS coupling, significantly reduced ROS level in both control and TAA homogenate supplemented with L-arginine (Figure 6).

### 2.6. Total NOS Activity

**Figure 7:**
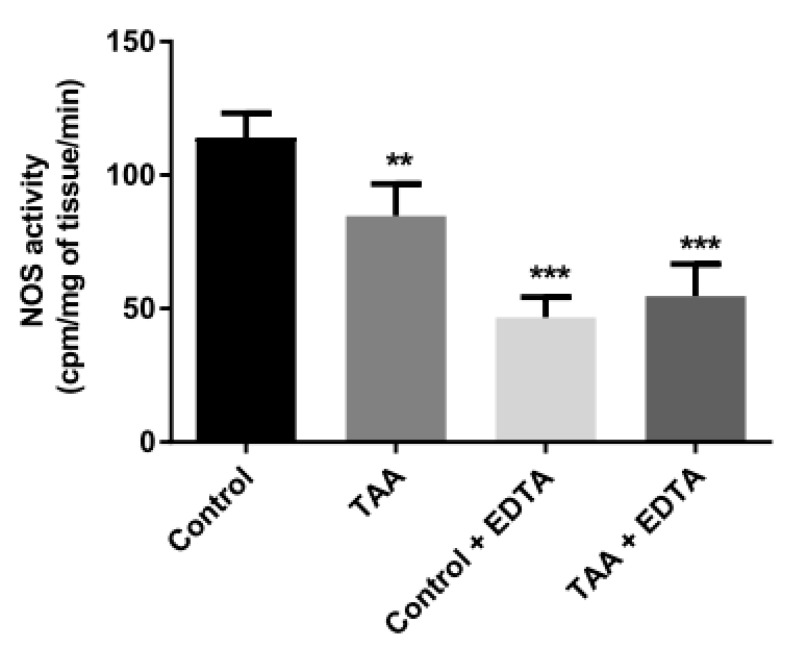
TAA reduces total NOS activity of rat brain prefrontal cortex Calcium ions chelation by EDTA additionally lowered NOS activity. Results are mean ± SD (n = 5). Significant difference vs. control ** *p* < 0.01; *** *p* < 0.001.

Total NOS activity was significantly reduced in the prefrontal cortex of TAA rats (Figure 7). Calcium ion chelation reduced the enzyme activity of control samples by ~70%, while also abrogating the difference between the activity of NOS in the prefrontal cortex of control and TAA rats. These results imply that (i) the majority of enzyme activity in the brain homogenate is attributable to eNOS and/or nNOS (Figure 7), (ii) TAA treatment does not affect the fraction of NOS activity which is attributable to iNOS.

### 2.7. CBF in the Prefrontal Cortex of TAA Rats 

**Figure 8:**
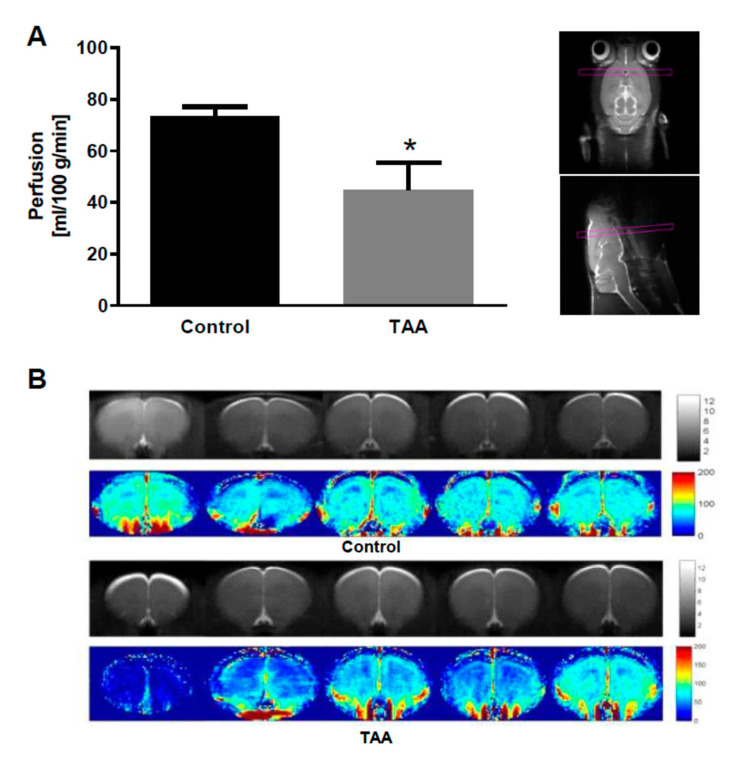
Magnetic resonance imaging (MRI; 3D ASL) analysis showed lowered cerebral blood flow (CBF) in the prefrontal cortex of TAA-treated rat. Pictures on the upper panel show a scanning plane (**A**). Below transverse plane MRI images of individual animal brains (in gray scale) and CBF (in color) (**B**). The red color labeling represents an increased CBF level, while blue indicates decreased CBF level. Significant difference vs. control * *p* < 0.05 (n = 5).

Given the well documented role of eNOS as a key regulator of microvascular tone, it appeared to be worthwhile to examine the effect of TAA treatment in the present model on CBF in the brain prefrontal cortex. MRI analysis revealed that TAA treatment reduced CBF by 40% (Figure 8), which can likely be reconciled with a decrease of eNOS expression (Figure 3) and its uncoupling (Figure 6).

## 3. Discussion

The key observation of the present study is that ALF significantly impairs brain eNOS function as a consequence of both eNOS protein loss and its uncoupling, as reflected by a drop in the ratio of the active dimer to the inactive monomer. The potential of eNOS dysfunction in L-arginine-evoked ROS formation was collectively deduced from the attenuation of ROS generation by a pan inhibitor of NOS, and from the observation that L-arginine-evoked enhancement of ROS generation was abolished by Ca2+ ions chelation, a maneuver abrogating eNOS activity. Of note, such conditions may evoke nNOS uncoupling as well. Recently, this phenomenon was reported in brain arteries of obese rats [28], and was suggested as a contributory factor in endothelial dysfunction during atherosclerosis in apolipoprotein E knockout (apoE-/-) mice [29]. It is worthy of note that both the regulation and functions of the constitutive NOS isoforms are largely determined by their subcellular localization, which is cell type-specific and mediated by various isoforms of caveolin. This interaction is crucial for the regulation of eNOS activity in vascular endothelial cells, where the enzyme is located at the luminal plasma membrane and changes its activity in order to modulate NO synthesis [30].

Few previous studies have specifically dealt with the fate of brain eNOS in liver failure; those that exist were confined to examinations of the consequences of chronic liver failure, and unanimously reported an increase of eNOS protein expression in the brain [14,31]. In the ALF model in which eNOS was likewise found to be increased, ALF was induced by a hepatic devascularization, a maneuver which induces liver dysfunction by a mechanism bypassing toxic damage [23]. The opposite responses to acute and chronic liver failure are best illustrated by differences in NO signaling-related cGMP status. Acute HE is accompanied by a NO-dependent, nNOS-mediated increase of cGMP [32,33], a status reflecting acute overexposure of the brain to ammonia [34], whereas in chronic HE, the cGMP level was depressed [35,36]. Selective regional effects of HE on the cGMP-synthesizing soluble guanylate cyclase, an enzyme synthesizing cGMP, may have contributed to the discrepancies in these findings [37]. 

The mechanisms responsible for changes in eNOS expression in the present ALF model remain to be determined. Regulation of eNOS is a complex phenomenon [38]. Apart from shear stress, classic eNOS inductors include VEGF [39], TGFβ [40], and H_2_O_2_ [41]. By contrast, pro-inflammatory factor TNFα [42] and LPS [43], and hypoxia [44,45], can reduce eNOS mRNA levels. Recently, the regulatory role of lncRNAs (STEEL and LEENE) and micro-RNAs (miR-92a; miR221/222) in eNOS level control was investigated [46,47,48]. Alternatively, posttranscriptional regulation may involve mRNA stability, methylation, or acetylation of histones H3, H4 [49]. Further, posttranslational control of eNOS involves mainly phosphorylation and protein–protein interactions, thereby fine-tuning eNOS activation positively or negatively [50,51]. All in all, further studies aimed at identifying the mechanisms by which eNOS responds to ALF will have to account for multitude metabolic steps at the levels of transcription, posttranslational modifications and protein–protein interactions.

The uncoupling of eNOS that directly generates O_2_^−^, and further, the formation of hydrogen peroxide and peroxynitrite, depend upon the availability of the NOS substrate, L-arginine, and NOS cofactors, in particular BH4. BH4 loss in the brain of TAA-treated rats, on one hand, and the attenuating effect of exogenously added BH4 on ROS production on the other hand, collectively appear to support the link between eNOS uncoupling and ROS formation in the ALF-affected brain. The involvement of endogenous L-arginine as an eNOS controlling factor appears less likely. The Km of L-arginine as an eNOS substrate is approximately 2.9 µM, whereas intracellular L-arginine concentration in the brain is in the mM range [52], rendering the system less vulnerable to alterations in endogenous L-arginine content. Of note, brain L-arginine concentration was unchanged in this ALF model [53], notwithstanding its elevation in the blood [54]. One other endogenous metabolite with a potential to modulate eNOS activity is NOSs endogenous inhibitor, asymmetric dimethylarginine (ADMA), a stable L-arginine derivative [52,55]. Indeed, the concentration of ADMA is elevated in the ALF-affected brain in the TAA model [53,56]; however, increased ADMA level was also found in chronic HE [14]. The nature of this modulation deserves further study.

Concerning BH4, a vicious circle appears to operate, whereby free O_2_^−^ oxidizes and inactivates BH4, and inversely, the uncoupled NOS isoform may contribute to O_2_^−^ generation in conditions where BH4 is deficient [57]. It has been shown that NO production by eNOS depends on the availability of intracellular BH4 [58]. The decrease of a brain BH4 evoked by ALF could be a consequence of altered BH4 synthesis pathway enzymes [59]. However, some evidence indicates that regulation of BH4 directly respond to changes in free radicals and nucleophilic oxidants such as hydrogen peroxide and peroxynitrite [60]. Cytochrome c, which was recently found to be elevated in a mouse ALF model [61], can also diminish BH4 content by direct oxidation [62].

In rat brain following focal ischemic incident, eNOS monomerization/uncoupling is coincident with decreased phosphorylation of its Ser1177 residue, even though a mechanistic coupling of the two phenomena remains to be proven [63]. In the future, it will be of interest to analyze the eNOS phosphorylation status in the ALF-affected brain.

As mentioned earlier, the uncoupling of eNOS in ALF-affected rat brain can be reconciled with, and is a likely contributor to, not only to the accumulation of ROS, but also to a number of subsequent pathogenic events. An ammonia-induced increase in ROS formation, including O_2_^−^ [8], promotes a marked reduction in central and peripheral NO bioavailability by rapid reaction with NO, leading to the formation of highly toxic and short-lived peroxynitrite anion [64,65]. Considerable evidence from in vivo and in vitro studies implicates, directly or indirectly, the role of tyrosine nitration in the evolution of HE symptoms, mainly brain edema [5,66,67,68]. Elevated brain protein nitration has been detected in the autopsy materials of HE patients with cirrhosis [69]. In turn, an increase in serum 3-nitrotyrosine, a direct product of proteins tyrosine residues nitration, is considered to be a biomarker in the diagnosis of patients with mild HE [27]. ALF in the present model likewise increases the nitration of brain protein tyrosine residues, which, at first sight, appears contradictory to the substantial decrease of total NOS activity and weakened eNOS function described here. However, these observations can be reconciled. Firstly, the accumulation of O_2_^−^ due to eNOS uncoupling appears to be sufficient to promote the increased formation of peroxynitrite and protein nitration [70]. Of note in this context, eNOS uncoupling-related O_2_^−^ generation and impaired NO signaling in cerebral microvessels were documented in GTP-cyclohydrolase I-deficient mice with BH4 loss [71]. In that study, the generation of 3-nitrotyrosine was significantly increased, whereas NO production and cGMP levels were reduced, pointing to the eNOS- independent NO molecules in diminished brain NO signaling. In turn, the subtle elevation of nNOS protein content in the brain of TAA-treated rats may be responsible for the intensified protein nitration. An elegant work carried on a mouse traumatic brain injury model using the genetic deficiency of NOS isoforms showed that nNOS, but not eNOS, is a trigger of enhanced brain protein nitration [72]. Moreover, in the brain of methamphetamine-intoxicated rats, tyrosine nitration was reported to be positively controlled by ADMA [73]. This mechanism may operate in the ALF-affected brain tissue with a concomitant increase of ADMA content [53,56], pointing to the role of nNOS in this chain of events. ADMA modulates NO production, and its effect on O_2_^−^ formation is BH4-dependent. In the presence of BH4, ADMA selectively inhibits O_2_^−^ generation from the enzyme. However, under conditions of nNOS depletion with BH4, ADMA no longer has any effect on O_2_^−^ [74]. Importantly, in the absence of BH4, ADMA increases eNOS-derived O_2_^−^ generation in a similar manner as the native substrate L-arginine, and the observed enhancement is of similar magnitude [75].

eNOS is critically involved in the maintenance of adequate cerebral blood flow (CBF) [76]. Therefore, deactivation of eNOS is a likely cause of the CBF impairment in the prefrontal cortex of TAA rats which was consistently observed in the present and in a previous study [77], as well as in studies in which different CBF recording procedures have been used [78]. Importantly in this context, several reports have implicated the participation of nNOS in CBF control (Santizo et al., 2000, Chi et al., 2003). The administration of a selective nNOS inhibitor, i.e., 7-nitroindazole (7-NI), depressed basal CBF in rats [79,80]. Moreover, under hyperbaric conditions, the increase in CBF in the cortex prior to the appearance of electrical discharges was likewise 7-NI-sensitive [81]. Therefore, further elucidation of nNOS-related CBF changes during ALF is required.

Even though CBF decrease is not a rule in ALF, a recent meta-analysis based on a representative bulk of studies confirmed that CBF decrease prevails in the majority of cases, irrespective of the causes of liver failure [82]. In this context, a decrease of cerebral perfusion resulting from the hepatic devascularization procedure precedes the increased intracranial pressure and brain edema [83]. Hence, if positively verified in other settings of ALF, the data obtained in the present study will support future considerations of brain eNOS as a contributor in the early stages of ALF.

In conclusion, we showed that the ALF induced in the TAA model resulted in a reduction of eNOS protein expression and in a decrease of the eNOS dimer/monomer ratio in the brain, but also in a partial loss of nNOS isoform. The study thus highlights the altered status of constitutive NOS isoforms as a potential contributor to brain impairment in ALF. In particular, the results show that (i) impaired expression/function of eNOS and/or nNOS, but not of iNOS, may be accounted for a decrease of NOS activity; (ii) eNOS-related aspects of brain redox status are related to the concurrent depletion of the eNOS cofactor, BH4; and (iii) impairment of eNOS is a likely contributor to reduced cerebral perfusion. Clearly, a detailed mechanistic analysis of the individual roles of eNOS and nNOS and their interplay in the above phenomena is merited in the future studies. 

## 4. Materials and Methods

### 4.1. Study Design, Acute Liver Failure Model and Biochemical Analysis

The studies were conducted on male Sprague–Dawley rats, outbred animal colony (Tac:Cmd:SD), supplied by the Animal House of Mossakowski Medical Research Centre, Warsaw, Poland, with initial body weights from 220 to 250 g, and kept under standard laboratory conditions at room temperature (22 °C) under an artificial light/dark cycle (12/12 h) with free access to standard laboratory food and tap water. All procedures were performed under the National Institutes of Health Guidelines for the Care and Use of Laboratory Animals and received approval 57/2015 (14 May 2015) from the 4th Local Ethics Committee for Animal Experimentation, Warsaw, Poland, as compliant with Polish Law (of 21 January 2005). All efforts were made to reduce the number of animals and to minimize their suffering. No special medication was given to reduce pain during experiments. The study complies with the ARRIVE guidelines for reporting animal research. No randomization was performed to allocate subjects in the study. This study was not preregistered.

Acute liver failure (ALF) was induced by three intraperitoneal (ip) injections of thioacetamide (TAA, Sigma-Aldrich, Saint Louis, MO, USA), (300 mg/kg body weight) at 24 h intervals [16]. Control rats were administered ip with the saline solution according to the analogical scheme. Animals were sacrificed 24 h after the last injection, with the onset of acute HE symptoms. Experiments were carried out on isolated livers and brain prefrontal cortices, using a key region for HE-dependent brain edema. Tissue samples were rapidly homogenized in a buffer appropriate to each protocol or frozen at −80 °C. The biochemical parameters of the blood plasma were evaluated: ammonia level as the main HE factor, activities of aspartate aminotransferase, alanine aminotransferase, and γ-glutamyl transpeptidase. Markers of liver damage were analyzed by a specialized veterinary laboratory. Additionally, liver sections derived from right lobes were fixed in PBS-buffered 4% paraformaldehyde solution and standard hematoxylin and eosin staining (block preparation in paraffin, section cutting 5–6 µm thick) were made to confirm liver damage. 

### 4.2. Protein Isolation, Western Blot, and eNOS Dimerization Analysis

The prefrontal cortex was homogenized in RIPA buffer containing Protease Inhibitor Cocktail (concentration 1:200, Sigma-Aldrich, Saint Louis, MO, USA), phosphatase inhibitor cocktail (concentration 1:100, Sigma-Aldrich, Saint Louis, MO, USA) and 50 μM sodium fluoride 0.5 M (Sigma-Aldrich, Saint Louis, MO, USA) and then centrifuged for 20 min at 15,000× *g* and 4 °C. The supernatants were transferred to a new Eppendorf tube. Equal amounts of protein (30 μg) were separated on 10% SDS–polyacrylamide gels and transferred onto a nitrocellulose membrane. Membranes were blocked with 5% nonfat dry milk in TBS-T buffer. The membranes were then incubated overnight with anti-nNOS (1:100, Santa Cruz Biotechnologies, Dallas TX, USA) anti-iNOS (1:250 Santa Cruz Biotechnologies, Dallas, TX, USA) anti-eNOS (1:250 Santa Cruz Biotechnologies, Dallas, TX, USA) and anti-Nitro-Tyrosine antibody (1:1000 Cell Signaling, Danvers, MA, USA) followed by 1 h incubation with HRP-conjugated-secondary IgG antibodies (1:5000, Sigma-Aldrich, Saint Louis, MO, USA) for detection by Clarity Western ECL Substrate (Bio-Rad Laboratories, Hercules, CA, USA). The first antibody was stripped off with 0.1 M glycine, pH 2.9, and second incubation was performed with an antibody against glyceraldehyde 3-phosphate dehydrogenase (GAPDH) 1 h incubation at 20–22 °C (1:7500, HRP-60004, ProteinTech, Manchester, UK). To determine eNOS dimmers content nonboiled lysates, low-temperature SDS-PAGE and nonreducing loading buffer (Thermo Fisher Scientific, Walhalm, MA, USA) were used. The chemiluminescent signal acquisition and densitometry analysis were conducted using the G-Box system (SynGene, Cambridge, UK) and GeneTools software (SynGene, Cambridge, UK). Total protein concentration was determined by the Lowry method using Modified Lowry Protein Assay Reagent (Thermo Fisher Scientific, Walhalm, MA, USA).

### 4.3. The Isolation of a Capillary Fraction 

Gray matter prepared from freshly isolated brains was homogenized in Ringer’s solution and centrifuged at 1500× *g* for 10 min at 4 °C. The pellet was resuspended in the same buffer, and centrifugation was repeated three times under the same conditions. The final pellet was homogenized in 10 mL of 0.25 M sucrose and centrifuged in a discontinuous sucrose gradient (0.25:1:1.5 M sucrose) (30,000× *g*, 30 min, 4 °C). The fraction containing microvessels obtained at the bottom of the tube was collected and transferred to a 0.2 mL plastic tube. 

### 4.4. Confocal Microscopy Visualization of eNOS

Isolated microvessels were fixed using 4% PFA for 30 min, and then washed three times in PBS and permeabilized/blocked for 2 h in 5% normal goat serum with 0.1% Triton X-100 at room temperature. Next, samples were washed with PBS and stained overnight using eNOS primary antibody (1:500 Cell Signaling, Danvers, MO, USA). After standard PBS washing, samples were stained with secondary antibody conjugated with AlexaFluor 488 (Invitrogen Corp., Carlsbad, CA, USA). Cell nuclei were contrastained with DAPI (Dako, Agilent Technologies, Santa Clara, CA, USA) for 15 min. Microvessel fraction was smeared on a microscopic slide and visualized using an Axio Observer Z.1 microscope confocal LSM 780 system (Carl Zeiss GmBH, Jena, Germany).

### 4.5. NOS Activity Measurement

NOS activity was determined using an NOS activity assay Kit (Cayman Chemical, Ann Arbor, MI, USA, #781001). Isolated prefrontal cortex tissue of rat brains was homogenized (1/5; *w*/*v*) in Assay Buffer, centrifuged (10,000× *g*, 15 min, 4 °C). The supernatant was used for NOS dependent conversion of 14C L-arginine to 14C L-citrulline, according to manufacturer’s protocol. To distinguish the activity of the iNOS (calcium-independent) and constitutive NOS (nNOS and eNOS), the calcium was chelated by EDTA. The radioactivity was measured in a Wallac 1409 Liquid Scintillation Counter (Perkin–Elmer, Waltharm, MA, USA). The NOS activity was expressed as CPM (counts per minute) of radiolabeled L-citrulline per gram of tissue per min.

### 4.6. Measurement of Nitrite and Nitrate 

The total nitrite and nitrate (NOx^−^) in brain prefrontal cortex and plasma was measured directly after tissue isolation using the Calbiochem Nitric Oxide Assay Kit (Merk, Darmstadt, Germany # 482655), which is a simple two-step process. The first step involves the conversion of nitrate to nitrite by the enzymatic action of nitrate reductase. The second step involves the addition of 2,3-diaminonaphthalene to convert nitrite to a fluorescent compound, i.e., 1 (H)-naphthotriazole. The fluorescence intensity was measured using an excitation wavelength of 375 nm and an emission wavelength of 450 nm using Fluorescence Microplate Reader FLUOstar OMEGA (BMG Labtech, Ortenberg, Germany).

### 4.7. BH4 Measurement

The concentration of BH4 in rat blood plasma and prefrontal brain cortex was measured using Rat BH4 Elisa Kit (MyBioSource, San Diego, CA, USA, #MBS2533582), according to manufacturer’s protocol for a 96-well plate assay. Brain prefrontal cortex was homogenized in ice-cold PBS (1/5; *w*/*v*) and centrifuged at 10,000× *g* for 5 min, before the supernatant was collected; 50 µL of freshly prepared tissue homogenate or 25 µL of EDTA plasma were used per plate well. The concentration of BH4 in the samples was then determined spectrophotometrically at a wavelength of 450 nm by comparing the OD of the samples to the standard curve (range 32.5–2000 pg. BH4/mL).

### 4.8. Reactive Oxygen Species Measurement

The total content of the ROS was determined fluorometrically using 5- (and-6)-carboxy-2′,7′-dichlorodihydrofluorescein diacetate (carboxy-H2DCFDA) fluorescent probe (Invitrogen). Isolated prefrontal cortex tissue was homogenized (1/5; *w*/*v*) in Locke buffer (154 mM NaCl, 5.6 mM KCl, 1.0 mM MgCl2, 2.3 mM CaCl2, 8.6 mM HEPES, 5.6 mM glucose, 0.1 mM glycine, pH 7.4) and diluted 10 times. Homogenate samples were incubated with NOS substrate L-arginine (100 μM) and/or L-NAME (100 μM), EDTA (1 mM), BH4 (100 nM) for 10 min, at 22–25 °C. The dose of L-arginine was chosen based on the literature and our previous study [77]. Subsequently, 10 μM carboxy-H2 DCFDA was added to each reaction mixture. Exactly 200 μL of the supernatant was used in the fluorescence intensity measurement at 485 nm excitation and 515 nm emission wavelengths (FLUOstar OMEGA, BMG Labtech, Ortenberg, Germany). The intensity values were directly proportional to the intracellular ROS content.

### 4.9. Cerebral Blood Flow Measurement

Arterial spin labeling MRI study was conducted 24 h after the last injection of TAA or saline at the Department of Magnetic Resonance Imaging, Institute of Nuclear Physics, Polish Academy of Sciences, Kraków. Experiments were performed exactly as described Czarnecka et al., 2018) using a 9.4 T (BioSpec 94/20 USR, Bruker, Germany) scanner. In brief, high-resolution anatomic images from the prefrontal cortex were acquired with spin-echo RARE (Rapid Acquisition with Relaxation Enhancement) sequence. For T1 relaxation time and tissue perfusion measurement, the FAIR-RARE (Flow-sensitive Alternating Inversion Recovery) sequence was used. Quantitative calculations were performed using an ad hoc Matlab (The MathWorks Inc., Natick, MA, USA) script.

### 4.10. Statistical Analysis

*t*-tests for unpaired samples were used to compare values between control and TAA groups with a Welch’s test for unequal variances. Statistical analysis of CBF was carried out by the two-sample Mann–Whitney U test for independent observations. Statistical differences among the means of more groups were evaluated by one-way ANOVA with a post hoc Dunnet’s test. All analyses were performed using 5.0 Graph Pad software (Graphpad Software Inc, La Jolla, CA, USA). Values were expressed as mean ± S.D. Significance was defined as *p* < 0.05 for all tests.

## Figures and Tables

**Table 1:** Rat model characteristics. Plasma level of ammonia (NH_3_) and activities of liver enzymes: aspartate aminotransferase (AST), alanine amino transferase (ALT), and gamma–glutamyl transpeptidase (GGTP). Initial, final rat body weights and rate of weight gain of control and TAA-treated rats (n = 5).

	Control	TAA
AST (GOT) (U/L)	142.3 ± 11.7	977.12 ± 124.38 ***
ALT (GPT) (U/L)	54.62 ± 2.03	325.41 ± 43.84 ***
GGTP (U/L)	2.65 ± 0.13	4.23 ± 0.33 **
NH_3_ (µmol/L)	64.54 ± 5.29	132.12 ± 18.64 **
Initial body weight (g)	208.5 ± 7.23	217.4 ± 8.1
Final body weight (g)	224.8 ± 9.47	196.1 ± 14.65
Body weight diff. (g)	16.3	−21.3

Abbreviation: AST, aspartate aminotransferase; ALT, alanine amino transferase; GGTP, gamma-glutamyl transpeptidase. Values are mean ± SD obtained as described in Materials and methods. *** *p* < 0.001, ** *p* < 0.01 vs. control group, n = 5.

## Data Availability

Data available on request.

## Abbreviations

ALF	Acute liver failure
BBB	Blood–brain barrier
BH4	Tetrahydrobiopterin
CBF	Cerebral blood flow
eNOS	Endothelial nitric oxide synthase
HE	Hepatic encephalopathy
NO	Nitric oxide
nNOS	Neuronal nitric oxide synthase
ONS	Oxidative nitrosative stress
ROS	Reactive oxygen species
TAA	Thioacetamide
TCA	Tricarboxylic acid
